# Transitions in metabolic and immune systems from pre-menopause to post-menopause: implications for age-associated neurodegenerative diseases

**DOI:** 10.12688/f1000research.21599.1

**Published:** 2020-01-30

**Authors:** Yiwei Wang, Aarti Mishra, Roberta Diaz Brinton

**Affiliations:** 1 Center for Innovation in Brain Science, University of Arizona, Tucson, AZ, 85721, USA

**Keywords:** Menopause, metabolism, inflammation, aging, neurodegenerative disease, hormones

## Abstract

The brain undergoes two aging programs: chronological and endocrinological. This is particularly evident in the female brain, which undergoes programs of aging associated with reproductive competency. Comprehensive understanding of the dynamic metabolic and neuroinflammatory aging process in the female brain can illuminate windows of opportunities to promote healthy brain aging. Bioenergetic crisis and chronic low-grade inflammation are hallmarks of brain aging and menopause and have been implicated as a unifying factor causally connecting genetic risk factors for Alzheimer’s disease and other neurodegenerative diseases. In this review, we discuss metabolic phenotypes of pre-menopausal, peri-menopausal, and post-menopausal aging and their consequent impact on the neuroinflammatory profile during each transition state. A critical aspect of the aging process is the dynamic metabolic neuro-inflammatory profiles that emerge during chronological and endocrinological aging. These dynamic systems of biology are relevant to multiple age-associated neurodegenerative diseases and provide a therapeutic framework for prevention and delay of neurodegenerative diseases of aging. While these findings are based on investigations of the female brain, they have a broader fundamental systems of biology strategy for investigating the aging male brain. Molecular characterization of alterations in fuel utilization and neuroinflammatory mechanisms during these neuro-endocrine transition states can inform therapeutic strategies to mitigate the risk of Alzheimer’s disease in women. We further discuss a precision hormone replacement therapy approach to target symptom profiles during endocrine and chronological aging to reduce risk for age-related neurodegenerative diseases.

## Introduction

The brain is the most energy-demanding organ in the body. In humans, the brain comprises 2% of total body mass yet consumes 20% of total oxygen and 25% of glucose ^1, 2^, making the brain susceptible to even modest disruptions in energy homeostasis ^3– 5^. Indeed, the aging brain, even of healthy aging individuals, is marked by glucose hypometabolism and mitochondrial dysfunction ^6, 7^. These metabolic and bioenergetic phenotypes are exaggerated in multiple age-associated neurodegenerative diseases (NDs), including Alzheimer’s disease (AD), Parkinson’s disease (PD), multiple sclerosis (MS), and amyotrophic lateral sclerosis (ALS) ^8– 18^.

Both preclinical and clinical studies reveal that alteration in brain metabolic status during aging and in ND is accompanied by shifts in energy sources, from glucose metabolism to fatty acid metabolism and ketone bodies ^19– 22^. While this strategy serves as an adaptation to sustain ATP production ^7^, it also leads to increased free radical production ^10, 23, 24^, lipid peroxidation ^21, 22, 25, 26^, oxidative stress ^27, 28^, and endoplasmic reticulum (ER) stress ^17, 18^. Increased production of damage-associated molecular patterns (DAMPs), such as extracellular ATP ^29^, mitochondrial DNA (mt-DNA), reactive oxygen species (ROS) ^30^, ceramides ^31^, oxidized low-density lipoproteins ^32^, and myelin debris ^21, 33– 35^, further induces chronic systemic inflammation. Induction of chronic systemic inflammation by metabolic stressors can serve as a missing mechanistic link from metabolic and bioenergetic dysfunction to ND ^36^.

In females, estrogen therapy initiated during the critical windows of peri-menopause to early menopause and surgical menopause has been shown to promote brain glucose metabolism ^37– 46^, reduce chronic inflammation ^47– 50^, and prevent cognitive decline ^51– 57^. Understanding the dismantling process of estrogen-regulated metabolic and immune systems during both chronological and endocrinological aging in the female brain can provide insights into ND prevention, diagnosis, and therapy. In this review, we discuss metabolic changes during pre-menopausal aging, peri-menopausal aging, and post-menopausal aging; their impact on neuroinflammation during each of the chronological and endocrinological transition stages; and the implications for NDs.

## Menopause and estrogen regulation of brain metabolism and inflammation

The menopausal transition is characterized by reproductive senescence and loss of ovarian hormones, particularly estrogen, in females. Estrogen regulates the systems of biology required for brain glucose metabolism and mitochondrial function ^38^. Estrogen promotes glucose uptake by both capillary endothelial cells of blood–brain barrier and neurons ^45, 46^, increases protein expression, and enhances activity of glycolytic enzymes ^41, 44^ and also increases protein expression of electron transport chain (ETC) subunits ^41– 43^. *In vitro* studies using rat embryonic neurons and glial cells also revealed increased maximal respiratory capacity in response to estrogen treatment ^58^. Not only can estrogen promote ATP production in healthy neurons *in vitro*, it can also preserve ATP production capacity in neurons exposed to Aβ1-42 ^59^. In surgically menopausal rodent models, estrogen treatment successfully prevented loss of mitochondrial respiratory capacity ^40^. Beyond promoting mitochondrial bioenergetics in the brain, estrogen can further reduce ROS production ^60^, promote calcium homeostasis, and protect cells from apoptosis ^61^, which collectively will promote mitochondrial function.

Decline in estrogen level during menopause is also associated with an increase in inflammation, marked by the increased expression of pro-inflammatory cytokines—interleukin-8 (IL-8), tumor necrosis factor alpha (TNF-α), IL-6, and interferon gamma (IFN-γ)—in response to T-cell activation ^47, 62, 63^. Sexual dimorphism, especially with the decline of estrogen, is particularly evident in the immune system ^64, 65^. Post-menopausal women have increased CD4/CD8 ratios and T-cell proliferation and activated T cell–mediated autoimmunity ^66, 67^. Multiple effects in the periphery are a direct response to the loss of immunosuppressive effects of estrogen. In the brain, estrogen is a master regulator of glucose metabolism, neuronal and glial bioenergetics, and microglial inflammation ^68^. The dysregulation of glucose metabolism in the brain is evident during the menopausal transition and can cause the accumulation of DAMPs, further causing the activation of innate and adaptive immunity to induce chronic low-grade inflammation ^36^.

Hormonal change associated with menopausal transition is a gradual process spanning multiple years, thus allowing adaptation in both metabolic and inflammatory function in the brain. Similarly, chronological aging before and after this endocrinological aging stage is coupled by systematic alterations in metabolic and immune systems. Below, we review these fluctuations in more detail during pre-menopausal, peri-menopausal, and post-menopausal stages.

## Chronological aging: prelude to endocrine aging

**Figure 1.**
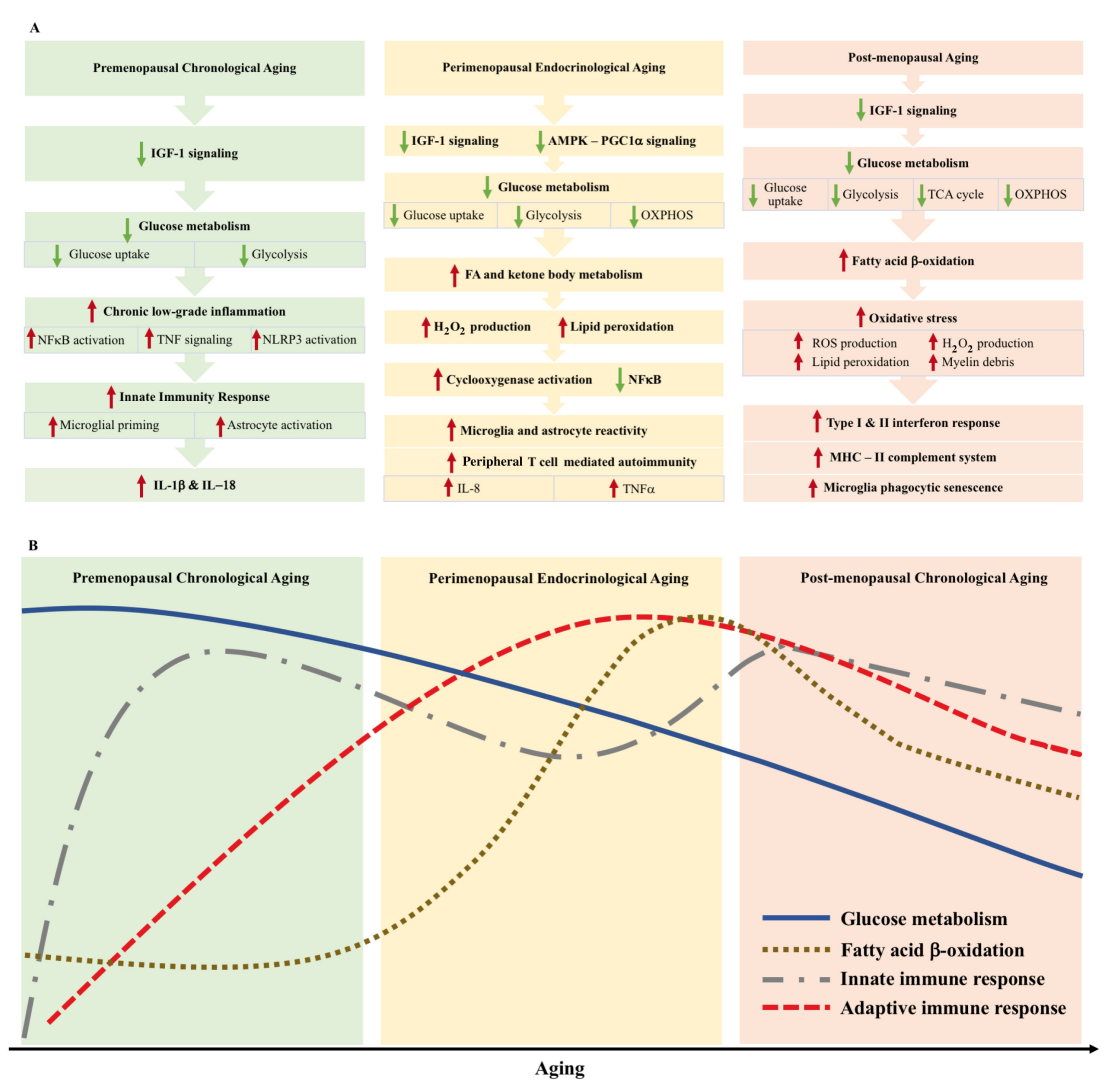
Metabolic and immune signaling during chronological and endocrinological transitions in the mid-life female brain. ( **A**) Summary of the transition in metabolic and inflammatory female aging in the brain. AMPK-PGC1α, AMP-activated protein kinase–peroxisome proliferator-activated receptor gamma coactivator 1-alpha; FA, fatty acid; H _2_O _2_, hydrogen peroxide; IGF-1, insulin-like growth factor 1; IL, interleukin; MHC, major histocompatibility complex; NFκB, nuclear factor kappa B; OXPHOS, oxidative phosphorylation; ROS, reactive oxygen species; TCA, tricarboxylic acid cycle; TNF, tumor necrosis factor. ( **B**) Temporal conceptualization of transitions in glucose metabolism, β oxidation, and innate and adaptive immune response during the course of female brain aging.

Aging is associated with a reduction in glucose metabolism and consequent increase in chronic low-grade inflammation ^69^ ( Figure 1). Clinical studies revealed that regional cerebral blood flow in mesial frontal cortex is negatively correlated with age in young to mid-life adults ^70^. Meta-analysis in adults between 20 and 50 years of age suggested that the reduction in brain glucose uptake was most likely due to a reduction in brain aerobic glycolysis ^71^. Similar findings were evident in a mouse model of the natural menopausal transition ^72^. In comparison with young female mice, mid-aged females had a significant reduction in brain glucose uptake, which was accompanied by significant down-regulation of neuronal glucose transporter 3 (GLUT3) and reduced glycolytic capacity, as evident by a significant reduction in hexokinase activity ^72^. Decline in glucose metabolic system in the brain was exacerbated in the triple-transgenic AD mouse model ^72^. Furthermore, aging from early to mid-adulthood in female rats was associated with significant down-regulation of both gene and protein expression of insulin-like growth factor 1 (IGF-1) in the hippocampus ^73^, suggesting that early disruption in insulin or IGF-1 signaling may underlie changes in brain glucose metabolism during this stage.

Decline in glucose metabolism was specific to the endocrine aging transition as comparable changes were not evident in the reproductively competent animals. No changes in the redox system, including total brain glutathione (GSH) level, GSH peroxidase activity, superoxide dismutase activity, and H _2_O _2_ clearance capacity, were observed in reproductively active female rats between early to mid-adulthood ^74^. Similarly, no significant changes were observed in brain synaptic mitochondrial total GSH, lipid peroxides, and cytochrome *c* oxidase levels in female mice between 10 and 24 weeks of age ^75^. These observations are expected given the relatively steady level of brain and plasma estrogen level during pre-menopausal aging.

Early indicators of disruption in glucose metabolism and IGF-1 signaling during the peri-menopausal phase are associated with increased inflammation through the activation of the inflammatory sensors of aging, nuclear factor-kappa B (NFκB) and TNF ^76^ ( Figure 1). In a peri-menopausal animal model (PAM), activation of NFκB pathway and TNF-related genes occurred during the chronological aging phase preceding the peri-menopausal transition. Activation of NFκB can also cause increased expression of Nod-like receptor pyrin domain-3 (NLRP3) inflammasome complex ^77^. The NLRP3 inflammasome complex is susceptible to an aging-related increase in insulin resistance and the onset of glucose hypometabolism during pre-menopausal aging ^78, 79^. The NLRP3 inflammasome complex is responsive to triggers such as age-associated DAMPs, including oxidized mt-DNA and extracellular ATP production due to the onset of metabolic dysfunction ^20, 21^, which initiate a cascade of chronic low-grade inflammation in the brain ^80^.

The two-step activation of NLRP3 inflammasome, which is an “immuno-metabolic sensor of aging”, leads to the priming of microglial cells ^81^. Secondary triggers such as extracellular ATP and mt-DNA cause the secretion of pro-inflammatory cytokines IL-1β and IL-18 ^82^. Interestingly, ketone body β-hydroxybutyrate mitigates the activation of NLRP3 inflammasome complex ^83^. Pre-menopausal aging is also associated with increased expression of complement genes in the hippocampus, where complement C4-A (C4A) acts as an upstream regulator ^20^.

Therefore, alterations in the metabolic profile in the brain can invoke an innate immune response from resident immune cells – microglia and astrocytes ( Figure 1). Simultaneous shifts in the metabolic phenotype lead to sustained chronic inflammatory responses, which when coupled with dysregulated steroidal hormone levels can exacerbate inflammation.

## Peri-menopause: metabolic-immunological transition

The peri-menopausal transition in females is defined by irregular menstrual cycles and decline in ovarian and brain estrogen production ^19, 84^. This endocrinological transition is associated with the early staging that dismantles estrogen regulation of brain bioenergetics ( Figure 1). Brain glucose uptake is gradually and significantly reduced during the peri-menopausal transition, especially in brain regions such as temporal lobe, precuneus, and frontal lobe, and is positively correlated with mitochondrial cytochrome oxidase activity ^7, 20, 85, 86^. As reviewed above, pre-menopausal aging is associated with decreased glycolysis but relatively unchanged oxidative phosphorylation, and mechanistic analyses in rat and mouse natural aging models recapitulating human menopausal transition revealed further reduction in glucose uptake as well as significant down-regulation of brain glucose transporters, key enzymes involved in glycolysis, and oxidative phosphorylation during the peri-menopausal transition ^20, 72^. Transcriptomic analysis revealed IGF-1 and AMP-activated protein kinase–peroxisome proliferator-activated receptor gamma coactivator 1-alpha (AMPK-PGC1α) signaling pathways as underlying regulators of metabolic changes ^20^. Brain glucose hypometabolism has also been described as a trigger of hot flashes in peri-menopausal females, an exaggerated compensatory neurovascular response to increase blood flow and glucose delivery to the brain ^87, 88^.

Estrogen promotes glucose metabolism in the brain, and loss of estrogen during menopausal transition can lead to utilization of auxiliary fuel sources in the brain, especially fatty acids and ketone bodies ^20– 22^. In natural menopausal mouse models, this was evident by the activation of cytoplasmic phospholipase A2 (cPLA2) in the brain ^21^, which was accompanied by increased brain mitochondria H _2_O _2_ production and lipid peroxide level ^21, 22^. Activation of cPLA2 and production of arachidonic acid are linked to increased inflammation through cyclooxygenase activation and increased prostaglandin and leukotriene secretion ^89^.

Linking these metabolic shifts to ovarian hormones was demonstrated in surgically menopausal rats as evidenced by increased brain lipid peroxidase level and decreased superoxide dismutase activity as well as significantly lower serum triglyceride but higher cholesterol, high-density lipids, and low-density lipids ^90^, a profile consistent with increased fatty acid metabolism. Increased mitochondria lipid oxidation may explain the accumulation of ROS during reproductive aging.

A causal link between metabolic dysregulation and consequent change in the inflammatory profile in the brain during peri-menopause has yet to be established. However, evidence suggests that the regulator of inflammation, nuclear factor kappa B (NFκB), is down-regulated in the hippocampus during peri-menopause ^20^. Meanwhile, in the periphery, T cell–mediated autoimmunity is worsened during peri-menopause and is associated with increased prevalence of rheumatoid arthritis, autoimmune hepatitis, and infectious disorders in women ^47, 66, 91^.

Decline in estrogen level during peri-menopause can also cause increased expression of adhesion molecules that participate in leukocyte transmigration ^47^. Regions such as the subventricular zone in rodent models that closely surround white matter tracts are particularly susceptible to the leukocyte transmigration ^92, 93^. Interestingly, autoimmune symptoms of MS, which generally manifests in early adulthood, are worsened during transition from peri-menopause to menopause ^94– 96^. Of note, peri-menopause is marked by significant up-regulation of pro-inflammatory cytokines secreted by CD4 T cells: IL-8 and TNF-α ^62, 63^. The occurrence of vasomotor symptoms such as hot flashes during peri-menopause has been correlated with increases in pro-inflammatory cytokines IL-8 and TNF-α ^62^. In contrast, circulating estradiol has an inverse relationship with serum IL-8 levels in peri-menopausal women ^63^. Microglial and astrocytic reactivities increase in response to declining estrogen. Surgical ovariectomy in animals caused increased expression of microglial markers CD14, CD11b, and CD45 and phagocytic markers Fcgr1 and Fcgr2b in the hippocampus and cortex ^97, 98^. Collectively, the metabolic-immunological transition of peri-menopause is a tipping point in age-related inflammation in recruiting adaptive responses to the brain ( Figure 1).

## Post-menopausal aging: profiles for risk and resilience ahead

Circulating and brain estrogen levels are at their lowest in post-menopausal females. Human and animal studies revealed that the brain becomes even less efficient in glucose metabolism and more reliant on lipid as its main fuel source ^7, 20– 22^. This is evident by reduced regional cerebral blood flow ^99^, brain glucose uptake and ketone body uptake ^100– 103^, glycolysis and citric acid (tricarboxylic acid cycle, or TCA) cycle enzyme activities ^22, 104, 105^, mitochondrial oxidative phosphorylation ^21, 22^, and increased enzyme activities of fatty acid β oxidation ^20– 22^ ( Figure 1). Surgical menopausal rodents also exhibit a higher fasting glucose level, greater brain insulin resistance, and impaired IGF-1 signaling ^26^. The hypothesis that the brain can catabolize its own white matter to generate free fatty acid to fuel itself is supported by high brain cytosolic phospholipase A2 activity, especially in the hippocampus, and accumulation of arachidonic acid in post-menopausal mice ^21^. This process causes an accumulation of myelin debris, a sterile inducer of inflammation ^21, 33^. Increase in myelin antigenic load thereby causes phagocytic senescence of microglia and can lead to dysregulated glial metabolism and alteration in extracellular matrix, causing an adaptive response from the periphery.

Meanwhile, oxidative stress accumulates in the brain, where reduced GSH level decreases, GSH disulfide (GSSG) level increases ^26, 106^, while ROS production such as H _2_O _2_ production and lipid peroxidation increases ^21, 22, 26^, which have been linked to further inflammatory activation of astrocytes and microglia ^107^ ( Figure 1). In the absence of the neuroprotective and anti-inflammatory effect of estrogen, ROS production together with accumulated sterile inflammatory triggers leads the female brain into a chronic inflammatory status ^107^. In ovariectomized rodent models, this is evident by increased expression of microglial reactivity markers – major histocompatibility complex class II (MHC II), CD74, CD86, CD68, and the complement system in the hippocampus and cortex ^97, 98^. This microglial molecular signature significantly overlaps with the “late-stage neurodegenerative disease” phenotype, which sees exacerbation of IFN response signaling, and overexpression of MHC genes ^108^. Together, these observations indicate that natural aging, particularly the menopausal transition, exhibits a phenotype of microglia that participates in neurodegeneration. It remains to be understood whether this molecular signature is a beneficial compensation or is the tipping point in the course of neurodegeneration.

## Implications for neurodegenerative diseases

The peri-menopausal transition is a tipping point for female brain aging ^7^. From the metabolic perspective, the process begins with decline in glucose metabolism ^7, 20, 22, 71, 72, 85, 104, 105^ and increase in insulin resistance ^20, 73^, followed by a compensatory mechanism to use fatty acids and ketone bodies as an auxiliary fuel source ^7, 20– 22^. Furthermore, this process is coupled with increased ROS production, oxidative stress, ER stress, and apoptosis ^10, 17, 18, 21– 28^, all of which provoke a neuroinflammatory reaction, to form a vicious circle that activates across metabolic crisis, oxidative and cellular stress, and chronic inflammation ^109^.

On the metabolic front, analysis of postmortem AD brains revealed significantly reduced activities of pyruvate dehydrogenase complex, isocitrate dehydrogenase, and α ketoglutarate dehydrogenase complex, whereas activities of succinate dehydrogenase and malate dehydrogenase were increased ^12^. ETC complex IV activity also declined ^110– 112^, as supported by reduced gene and protein expression of complex IV subunits ^113– 115^. Similarly, patients with PD have reduced resting-state glucose metabolism in the brain, especially in cortical regions and motor networks ^85, 116, 117^, and mitochondria from cultured PD neurons also demonstrated reduced ETC activities ^118, 119^. Patients with MS have axonal degeneration and oligodendrocyte dysfunction ^120– 122^, which have also been attributed to mitochondrial bioenergetic deficiency in neurons and oligodendrocytes ^122– 124^ and excessive ROS production ^121, 122, 125^. Patients with ALS have increased energy expenditure ^126^ accompanied by impaired glucose tolerance ^127^, increased insulin resistance ^128^, and hyperlipidemia ^129^.

Decline in neuronal glucose metabolism and mitochondrial function can serve as an initiating factor for chronic inflammation. Microglial stress response as observed during aging and neurodegeneration is seen through the accumulation of DAMPs due to metabolic dysregulation ^130^. Sterile inducers of microglial inflammation set in motion chronic low-grade inflammation, which leads to premature microglial senescence and excessive synaptic pruning. Specifically, single-cell RNA-sequencing (RNA-seq)-based studies on familial AD models and ALS animal models indicated a disease-associated microglia (DAM) phenotype that is different from that of homeostatic microglia ^131, 132^. DAM is characterized by up-regulation of TREM2, APOE, TYROBP, ITGAX, and B2M and down-regulation of CX3CR1, P2RY12, and TMEM119 gene expression ^132^. The shared phenotype of this microglial subpopulation between AD, ALS, and normal aging indicates that a microglial subpopulation dedicated to debris clearance and combating neurodegeneration emerges in the brain.

Engagement of the complement system and phagocytosis are fundamental to synaptic pruning during development, yet dysregulation of this system can lead to excessive loss of synapses ^133^. Dysregulation in complement signaling mediated through complement receptor 3 (CR3) has been implicated in a rotenone-induced PD mouse model ^134^. Microglial ablation achieved by blocking colony-stimulating factor 1 receptor (CSF1R) signaling without reducing amyloid-β load in the brain was beneficial in restoring behavioral deficits and synaptic function ^135, 136^. While microglial ablation leads to complete loss of microglia (including homeostatic and DAM microglia), regulation of inhibitory checkpoint signals such as CX3CR1 that play a prominent role in DAM expansion could be pivotal to the development of ND therapeutic strategies ^131^. Activation of inflammasome complex such as NLRP3 and NLRC4 also contribute to increased pro-inflammatory cytokine secretion and increased amyloid-β load in AD mouse models ^131^.

Dysregulation of IFN signaling is central to MS pathology and demyelination ^137^. Interferonopathy induced by USP18 down-regulation increases microglial reactivity associated with white matter tracts to cause demyelination ^138, 139^. Up-regulation of type I and type II IFN response genes and MHC II has also been documented as a late-stage disease response in animal studies that model progressive neurodegeneration and aging ^108^.

During the peri-menopausal transition, we identified the emergence of a bioenergetic and inflammatory phenotype that is shared between neurodegenerative disorders. Therefore, therapeutically targeting the metabolic and immune profiles that emerge during this transition state could potentially limit the development of at-risk phenotypes for age-related NDs.

## Genetic factors for neurodegenerative disease risk

Over the past decades, it became increasingly clear that genetic variances modulate metabolic and inflammatory phenotypes present in at-risk populations and patients with ND. For example, apolipoprotein E (APOE) genotype, particularly APOE4, is a widely recognized risk factor for AD ^140– 149^. APOE4 carriers not only have lower brain glucose uptake compared with non-carriers ^150– 155^ but also exhibit more severe, more widespread, and more rapid decline in brain glucose hypometabolism ^150, 153, 156– 158^.

Mechanistic studies indicate an association between APOE4 genotype and mitochondrial dysfunction and glucose hypometabolism in the brain ^150, 151, 153, 156, 158– 163^. APOE4 gene expression in humans was associated with down-regulation of genes involved in mitochondrial oxidative phosphorylation and energy metabolism ^164, 165^. In APOE4 knock-in mice, proteomic analysis revealed decreased expression of proteins involved in the TCA cycle, glucose, lipid and amino acid metabolism ^166^.

The impact of metabolic health on cognitive function was investigated in a cohort of healthy post-menopausal females ^167, 168^. Outcomes of these analyses indicated that a metabolic profile indicative of risk for metabolic syndrome/type 2 diabetes was associated with significant deficits in verbal memory, executive function, and global cognitive performance, which were more prominent in APOE4 carriers ^167, 168^.

Microglia and astrocytes contribute as major cell types in the production of APOE; therefore, the contribution of APOE to innate immune responses can be expected ^169– 173^. Up-regulation of APOE expression as part of the DAM phenotype contributes to a microglial phenotype that combats progression of disease phenotype ^132^. Given that the APOE4 allele is considered evolutionarily conserved to protect against viral and bacterial infections, in mouse models of familial AD with the APOE4 risk factor, inflammatory challenges such as lipopolysaccharide (LPS) induced a robust pro-inflammatory reaction ^172, 174, 175^. APOE4 interferes with microglial clearance function through the down-regulation of insulin-degrading enzymes ^176, 177^ and neprilysin ^178^ which further exacerbates accumulation of DAMPs such as amyloid-β and activation of the innate immune response ^36^. These mechanistic findings are indicative of the increased chronic low-grade inflammation clinical profile seen in human APOE4 carriers, who have increased expression of C-reactive protein and reduced latency to the onset of AD ^179^.

On the therapeutic side, APOE4-positive patients with mild-to-moderate AD were less responsive to rosiglitazone, which can improve mitochondrial efficiency and glucose metabolism ^180, 181^. Interestingly, APOE4 carriers exhibit a better response to non-steroidal anti-inflammatory treatment ^169, 182^. Inflammation burden-specific treatment for APOE4 carriers will be critical for the development of APOE4 targeted AD therapeutics ^169^.

## Precision treatment strategy and hormone therapy

Given the impact of genetic variance on phenotypes of aging, metabolism, and inflammatory profiles, a personalized precision medicine approach that takes into consideration differences in genetic background, stage of endocrinological/chronological aging, and timing of treatment should be considered when designing future prevention or intervention strategies to promote healthy brain aging in females.

Understanding how the menopausal metabolomic-immuno-crisis drives risk of NDs in females offers insight into prevention and treatment strategies targeted to each chronological and endocrinological aging stage. Furthermore, identification of the subset of females at higher risk for NDs is pivotal to a precision medicine approach for healthy brain aging. Clinical studies have suggested that the combination of APOE genotype and metabolic phenotype can help identify post-menopausal females at risk for cognitive decline ^167, 168^.

The data indicate that, during the transition from peri-menopause to menopause, the metabolic-immune systems are in transition from a brain fueled by glucose metabolism to a brain fueled by auxiliary lipid and fatty acid metabolism that generates ketone bodies. This shift in fuel source is mediated in large part by a parallel and interacting shift from an innate immune phenotype to an activated and pro-inflammatory adaptive immune phenotype.

Three key issues for precision hormone therapy require consideration. The first is the limited time window for efficacy of hormone therapy. The introduction of hormone therapy as a preventive versus a treatment intervention has limited windows of efficacy. Efficacy of hormone therapy is limited to when the system is undergoing a transition from peri-menopause to menopause ^7, 53– 56, 183– 190^. Hormone therapy has limited to no efficacy and is not advised in late post-menopause for either natural or surgical menopausal females ^57, 183– 188, 190, 191^. Second, therapeutics should target the metabolic and immune systems of biology rather than single components within these complex systems. Third, hormone or other therapeutics should specifically target stage-specific metabolic and immune signaling pathways ( Figure 1). Hormone therapies, particularly estrogen and progesterone, are regulators of systems of biology that promote glucose metabolism and repress inflammatory processes, which can address these issues ^38, 40, 68, 192– 194^.

These considerations are born out in studies of early menopausal females in which those receiving estrogen replacement therapy had higher brain glucose uptake, regulated insulin signaling, and sustained cognitive function ^51, 53– 56, 195– 197^. In animal studies, estrogen immediately following ovariectomy resulted in improved bioenergetic capacity, insulin resistance, increase antioxidants, and reduced lipid peroxidation relative to untreated animals ^40– 44, 198, 199^.

Use of hormone therapy or estrogen replacement therapy can also mitigate menopause-related neuroinflammation. Estrogen mitigates the inflammatory action of sterile and infectious agents on microglia and astrocytes by down-regulating inducible nitric oxide synthase (iNOS) and cyclooxygenase-2 (COX-2) expression, reducing TNF-α, IL-1β, macrophage inflammation protein-2 secretion, and ROS production ^200^. Estrogen mediates its effect through both intracellular estrogen receptors ERα and ERβ, which are abundantly expressed in microglia and astrocytes. Ovariectomizing rodents increases microglial reactivity and changes the morphology to a pro-inflammatory phenotype ^200^. Preventive estrogen treatment before ovariectomy mitigates the development of pro-inflammatory phenotype of microglia by down-regulating complement and microglial reactivity genes ^98^. Peripheral immune cells also respond to hormone therapy through mitigating pro-inflammatory responses seen during menopause and preventing immune senescence by maintaining lymphocytes and monocyte numbers ^36^.

Collectively, the data indicate that hormone therapy initiated early in the menopausal transition results in sustained brain metabolic viability and prevention of age-related chronic low-grade inflammation and subsequent development of adaptive immune responses related to inflammation and autoimmunity.

## Conclusions

Herein, we reviewed metabolic and inflammatory profiles that emerge during female chronological and endocrinological brain aging. Furthermore, analysis of data from a broad range of studies and laboratories indicates that metabolic and immune transitions in the brain are linked to act in concert. The pre-menopausal aging phase is characterized by a decline in glycolysis and glucose metabolism and a rise in innate immune responses. Estrogen dysregulation sets the stage for peri-menopause and causes further decline in glucose metabolism and mitochondrial oxidative phosphorylation. Disruption in estrogen regulation causes an increase in T cell–mediated adaptive responses. During the post-menopausal aging phase, to offset the bioenergetic demand of neurons, the shift from utilization of glucose to the utilization of auxiliary fatty acid fuel sources to generate ketone bodies results in myelin breakdown. Accumulation of myelin debris induces a rise in the IFN response and MHC expression. Parallels to metabolic and immune profiles comparable to those of the prodromal phases of AD and MS emerge during pre- to peri- to post-menopause aging transition. Biomarkers of risk for post-menopausal age-associated ND coupled with biomarkers of therapeutic efficacy remain to be integrated with hormone therapy interventions. In the twenty-first century, precision hormone therapy is feasible given the current technologies and knowledge of menopausal brain health.
